# Application of Multilayer Evidence for Annotation of C-Terminal *BRCA2* Variants

**DOI:** 10.3390/cancers13040881

**Published:** 2021-02-20

**Authors:** Henriett Butz, János Papp, Anikó Bozsik, Lilla Krokker, Tímea Pócza, Edit Oláh, Attila Patócs

**Affiliations:** 1 Department of Molecular Genetics, National Institute of Oncology, H-1122 Budapest, Hungary; butz.henriett@med.semmelweis-univ.hu (H.B.); janos.papp@oncol.hu (J.P.); bozsik.aniko@oncol.hu (A.B.); timi.pocza@gmail.com (T.P.); e.olah@oncol.hu (E.O.); 2 Hereditary Cancers Research Group, Hungarian Academy of Sciences-Semmelweis University, H-1089 Budapest, Hungary; krkkr.lilla@gmail.com; 3 Department of Laboratory Medicine, Semmelweis University, H-1089 Budapest, Hungary

**Keywords:** *BRCA2*, breast cancer, ovarian cancer, p.K3326*, cancer predisposition, NGS

## Abstract

**Simple Summary:**

The potential pathogenic role of germline *BRCA2* c.9976A>T and c.10095delinsGAATTATATCT was evaluated in hereditary breast and ovarian cancer (HBOC) patients by investigating 2491 probands and verified in an independent cohort of 122,209 patients. Although the c.10095delinsGAATTATATCT variant was more prevalent among patients compared to control populations, no increased risk for cancer was found. No association between c.9976A>T and clinicopathological parameters or elevated risk for HBOC cases was detected. However, lung cancer was more prevalent in families carrying c.9976A>T compared to pathogenic *BRCA1/BRCA2* carrier families. An increased frequency of pancreatic cancer was found in families where c.9976A>T occurred together with other pathogenic *BRCA1* variants. The C-terminal stop codon variants showed no association with other pathogenic *BRCA2* variants. No loss of heterozygosity (LOH) in tumor tissue and no allelic imbalance in RNA level were confirmed. The c.9976A>T variant may be considered as a potential risk for lung cancer, and a potential modifying factor in pancreatic cancer when it occurs along with the pathogenic *BRCA1* variant, although this observation should be validated in a larger sample cohort.

**Abstract:**

The clinical relevance of the *BRCA2* C-terminal stop codon variants is controversial. The pathogenic role of the germline *BRCA2* c.9976A>T and c.10095delinsGAATTATATCT variants in hereditary breast and ovarian cancer (HBOC) patients was evaluated. An association with clinicopathological parameters was performed in 2491 independent probands diagnosed with HBOC and in 122,209 cancer patients reported earlier. Loss-of-heterozygosity (LOH) in tumor samples and allelic imbalance in RNA extracted from peripheral blood cells were investigated. Neither c.10095delinsGAATTATATCT or c.9976A>T variants showed significant association with clinicopathological parameters or elevated risk for HBOC-associated tumors. Lung cancer was more prevalent in families carrying the c.9976A>T variant compared to pathogenic *BRCA1* or *BRCA2* carrier families. An increased prevalence of pancreatic cancer was found in families where c.9976A>T occurred together with other pathogenic *BRCA1* variants. An increased risk for familial pancreatic, lung and upper aero-digestive tract cancers was confirmed in the validation set. Regarding *BRCA2* C-terminal variants, no linkage with other pathogenic *BRCA2* variants, no LOH in tumor tissue and no allelic imbalance in RNA level were confirmed. The c.9976A>T variant may be considered as a potential risk for lung cancer, and a potential modifying factor in pancreatic cancer when it occurs along with the pathogenic *BRCA1* variant, although this observation should be validated in a larger sample cohort.

## 1. Introduction

In the American College of Medical Genetics and Genomics (ACMG) classification system stop codon (truncation) variants are usually considered to be pathogenic/likely pathogenic [1]. Additionally, stop codon variants of the *BRCA2* gene are frequent among all pathogenic variants, leading to a significant increase in the risk of breast and ovarian cancer. However, damaging variants of the C-terminal of the *BRCA2* gene have not been investigated or are not considered pathogenic due to Evidence-based Network for the Interpretation of Germline Mutant Alleles (ENIGMA) classification [2]. Among their criteria, they suggest that “a variant predicted to disrupt expression only of protein sequence downstream of position 3325 would be considered unlikely to be clinically important. Further functional and clinical studies are underway to refine risk, if any, for predicted nonsense or frameshift variants downstream of position 3326”. The *BRCA2* protein has multiple roles besides the well-known DNA double-stranded break repair by homologous recombination, such as maintaining genome stability, including DNA replication, telomere homeostasis and cell cycle progression [3]. These functions have been investigated by different assays but not all functions are available for exploration due to either technical or study limitations. The C-terminal of the *BRCA2* protein contains interaction sites of RAD51 and multiple phosphorylation sites affecting their function [4,5,6]. While, among these, S3291 probably has the highest impact in the *BRCA2*–RAD51 interaction, a protein sequence of amino acids 3265–3330 of the *BRCA2* protein was also reported to bind RAD51 [4,5,6]. Additionally, a serine at the position of 3397 located more terminally from c.9976A>T (K3326*) is also a phosphorylation site, the function of which has been poorly investigated (https://www.phosphosite.org (accessed on 30 December 2020)). Furthermore, the interaction of the *BRCA2* C-terminal with RAD51 may be less significant for homology-directed repair (HDR) than for the protection of stalled replication forks, a relatively newly discovered and HDR-independent function of *BRCA2* [7]. Stalled replication fork degradation occurs due to MRE11 nuclease in the lack of *BRCA2*-mediated fork protection, in which its C-terminus has an essential role [7]. Although the defect of this function of *BRCA2* did not lead to cell survival change, the frequency of chromosomal aberrations was found to be increased [7]. It was suggested that *BRCA2* protein defective in maintaining fork stability and still proficient in HDR would be insensitive to Poly (ADP-ribose) polymerase (PARP) inhibitors, which specifically exploit the defect of double-strand repair [7].

Previous literature data regarding the clinical relevance of *BRCA2* c.9976A>T C-terminal stop codon variants have remained controversial, suggesting either a potential pathogenic role [8,9,10] or no clinical significance [11,12,13]. The *BRCA2* c.9976A>T variant results in a stop codon at amino acid position 3326. Initially, it was considered pathogenic due to its nonsense coding nature, however, it was reclassified as non-pathogenic based on case–control studies [11]. Among previously published literature, breast cancer risk was elevated for *BRCA2* c.9976A>T carriers when compared to a control population in three reports [9,14,15]. Studies investigating only ovarian cancer patients, except of Stafford et al. (2017) [16], showed similar odds ratios (ORs). Interestingly, among familial pancreatic, lung and upper aero-digestive tract (UADT) cancer patients, c.9976A>T carrier status was associated with increased risk for developing cancer [8,10,17].

Using basic classification rules, the c.10095delins GAATTATATCT variant, due to its nature (a combination of a deletion and an insertion leading to frame shift and consequently a premature stop codon, Figure S1), can be regarded as pathogenic. However, due to its localization (terminal from 3326 position), it is usually considered as a benign variant. Indeed, in ClinVar database, 11 of the 15 entries interpreted this variant as benign/likely benign and four submitters considered it as a variant with unknown significance (VUS). Additionally, of the 11 studies reporting c.10095delins GAATTATATCT, the vast majority considered it as a VUS [18,19,20] or clinically not important [21] in breast/ovarian cancer patients, and a VUS in familial pancreatic cancer [22], while it was interpreted as benign in ovarian cancer patients [23]. Interestingly, in a study prioritizing variants in hereditary breast and ovarian cancer genes in patients lacking known *BRCA* mutations, the c.10095delins GAATTATATCT variant was categorized as likely pathogenic based on co-segregation analysis (likelihood ratio 3.71) [24].

Therefore, the aim of our study was to investigate the prevalence of *BRCA2* C-terminal stop codon variants among our breast/ovarian cancer patients sent to germline *BRCA1/2* gene testing and their co-segregation with clinicopathological parameters and study the loss of heterozygosity and allelic imbalance. An extensive literature review of an additional 122,209 cancer patients was also performed to assess the effects of the c.9976A>T variant on cancer risk.

## 2. Results

### 2.1. Frequency and Characteristics of BRCA2 Terminal Stop Codon Variants in Breast Cancer Patients

**Figure 1:**
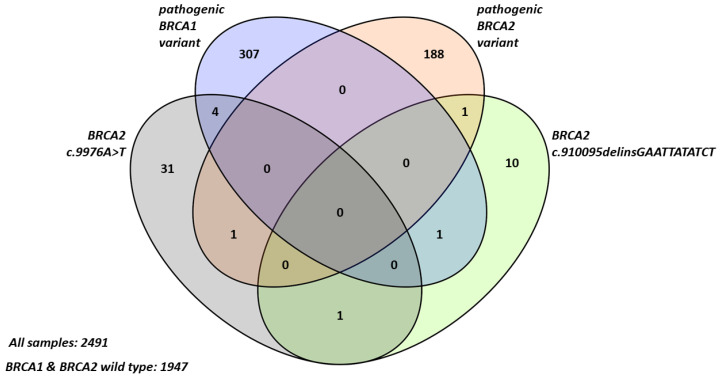
Numbers of *BRCA1* and *BRCA2* variants identified in our cohort.

Out of 2491 independent breast/ovarian cancer patients, c.9976A>T and/or c.10095delinsGAATTATATCT stop variants were identified in 49 cases (Figure 1). Among 49 cases, c.9976A>T was detected in 36, c.10095delinsGAATTATATCT in 12 cases and c.9976A>T together with c.10095delinsGAATTATATCT in 1 case. These variants co-occurred with other pathogenic *BRCA1* or *BRCA2* variants (c.9976A>T in five cases (5/37: 13.51%) and c.10095delinsGAATTATATCT in two cases (2/13: 15.38%)) (Table 1). The frequency of double heterozygosity in the investigated population was low: 0.002 (5/2491) and 0.0008 (2/2491) for c.9976A>T and c.10095delinsGAATTATATCT, respectively. Pathogenic *BRCA1* with a pathogenic *BRCA2* variant in the same patient has not been identified in our cohort (Figure 1).

Age of disease appearance did not differ in terminal stop codon variant carriers compared to pathogenic *BRCA2* carriers or *BRCA1/2* wild type patients (see Table 2). The Ki67% proliferation index, the prevalence of triple negative breast cancer (TNBC) or multiplex HBOC were increased only when the c.9976A>T variant accompanied a pathogenic *BRCA1* variant (Table 2).

### 2.2. Familial Cancer Prevalence in Probands with BRCA2 Terminal Stop Codon Variants

#### 2.2.1. Hereditary Breast and Ovarian Cancer (HBOC) Syndrome-Related Cancers

To assess the effects of the c.9976A>T and c.10095delinsGAATTATATCT variants on cancer risk, standard familial data were obtained from all probands (see Methods). Accordingly, “strong familiarity” was defined by the presence of breast/ovarian cancer before 50 years and/or male breast cancer in the family. Additionally, cases were categorized as “syndromic” when HBOC-related tumors (breast, ovarian, male breast, prostate or pancreatic cancer) occurred in the family of the proband (Table S1).

Expectedly, regarding HBOC-related tumors in the family of probands carrying a pathogenic *BRCA1/2* variant, breast and ovarian cancer were more frequent before the age of 50 years, and irrespective of age as well. The prevalence of HBOC-related tumors did not differ in families of c.9976A>T or c.10095delinsGAATTATATCT carrier probands as compared to *BRCA1/2* wild type patients (Table 2).

**Figure 2:**
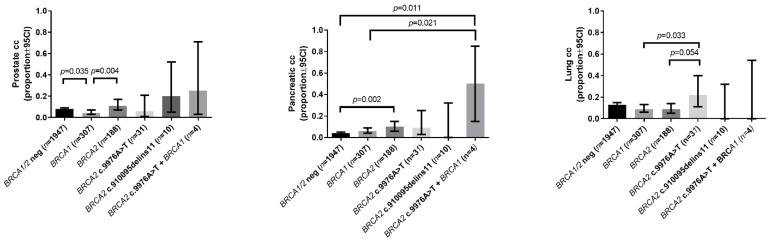
Prostate, pancreatic and lung cancer prevalence in HBOC probands’ families (proportions with ± 95%CI).

Pancreas and prostate cancers were more common in families with pathogenic *BRCA2* variant carrier probands (Table 2, Figure 2). Interestingly, pancreas cancer was also more frequent in double heterozygotes of a *BRCA1* pathogenic variant and *BRCA2* c.9976A>T variant, suggesting a potential genetic modifier effect (Table 2, Figure 2). The statistical power of this comparison was 78.3%.

Strong familiarity and syndromic familial history were characteristic only for the families of pathogenic *BRCA1/2* variant carriers, while families of probands carrying only the *BRCA2* C-terminal stop codon did not differ from *BRCA1/2* wild type patients’ families regarding HBOC syndrome-related tumor types (breast, ovarian, prostate and pancreatic cancer) (Table 2). In addition, pancreatic cancer occurred more frequently in families carrying a pathogenic *BRCA1* variant along with *BRCA2* c.9976A>T (double heterozygotes) (0.5) compared to wild type (0.04) and pathogenic *BRCA1* carrier families (0.06) (Table 2, Figure 2).

#### 2.2.2. Prevalence of Other Cancers

We assessed the frequency of lung, skin, head and neck, hepatocellular and gastric cancer in our cohort. We observed that lung cancer was more common in families of *BRCA2* c.9976A>T carrier probands (0.22) when compared to *BRCA1/2* wild type (0.13) or pathogenic *BRCA1* (0.09) or *BRCA2* (0.09) variant carrier families (Table 2, Figure 2). We did not find significant differences in the occurrence of head and neck, gastric or hepatocellular cancer in our families (Table 2, Table S2).

### 2.3. Functional Evaluation of the Potential Pathogenicity of BRCA2 C-Terminal Stop Codon Variants (Loss Of Heterozygosity, Allelic Imbalance, Minor Allele Frequency)

Loss of heterozygosity (LOH) in the tumor sample of a variant carrier is considered as supporting evidence for pathogenicity according to the ACMG guidelines. Therefore, we tested LOH in c.9976A>T carrier cases where c.9976A>T occurred without any other pathogenic *BRCA1* or *BRCA2* variant and where a tumor sample was available. In 26 tumor tissue–blood (somatic vs. germline) pairs, we did not confirm LOH in any of the tumor specimens.

We also investigated if C-terminal stop codon variants influence allelic stability in three samples. By cDNA sequencing, we did not find difference in allelic expression between wild type and variant-carrier strands in c.9976A>T nor c.10095delinsGAATTATATCT cases.

Variant segregation with disease phenotype supports pathogenicity. As, currently, c.9976A>T and c.10095delinsGAATTATATCT are not considered obviously pathogenic variants for HBOC cancers, healthy family members could not be screened for this variant within our national genetic counseling system.

Regarding allelic frequency, we observed a minor allele frequency (MAF) of c.9976A>T as 0.0074 in our proband population that did not differ significantly from those reported for European non-Finnish samples or total MAFs (0.008723 and 0.006468, respectively) based on the gnomAD database.

Determining the prevalence of *BRCA2* c.10095delinsGAATTATATCT was challenging because it is a complex variant (a combination of a deletion and insertion), therefore, in different databases, it appears as two distinct variants (e.g., in the gnomAD database: c.10094_10095insGAATTATAT and c.10095_10096insT, leading to frameshift and a premature stop codon), due to different variant-calling algorithms used during variant annotation of next generation sequencing data. (The correct description of the variant is c.10095delinsGAATTATATCT according to Human Genome Variation Society (HGVS) nomenclature.) Accordingly, we found that the *BRCA2* c.10095delinsGAATTATATCT variant was more prevalent among our patients compared to the control population (0.00261 vs. 0.00047, respectively).

Recently, Higgs et al. reported multiple co-occurrences of the *BRCA2* c.9976A>T variant with the pathogenic *BRCA2* c.6275_6276delTT (p.(Leu2092ProfsTer7)) frameshift variant in 52 families, while only 1.3-1.7% of the patients carried the *BRCA2* c.9976A>T variant alone [7]. Therefore, we investigated if these variants are in linkage in our patient cohort. Surprisingly, we did not detect the *BRCA2* c.6275_6276delTT variant in our sample set (2491 probands) at all, including all patients carrying *BRCA2* c.9976A>T (Table 1).

### 2.4. Re-Analysis of BRCA2 c.9976A>T and c.10095delinsGAATTATATCT Variants by Re-Analysis of All Published Data Where These Variants Were Investigated

Due to its indistinct annotation, still, there are literature data regarding the *BRCA2* c.10095delinsGAATTATATCT variant (Table 3). Allelic frequency is observed in a wide range (0.00099–0.03846), but in all data sets, it was less than 0.04. Most of the reports interpreted the variant as having unknown significance, but in ovarian cancer, it was described as a benign variant (Table 3).

Because of the controversial literature data regarding the clinical relevance of the *BRCA2* c.9976A>T variant, we conducted an extensive literature search and collected all available data. We found 38 studies reporting 122,209 cases investigating *BRCA2* gene variants in different cancer types including breast, ovarian, pancreatic, lung, upper aero-digestive system, urinary tract and skin cancers (Table 4). *BRCA2* c.9976A>T was available for evaluation in 115,854 cases. Carrier status was reported in 5129 patients of the 115,854 cancer cases. The average minor allele frequency (MAF) of the variant in breast/ovarian cancer patients was 0.0096. Regarding breast cancer cases, the average MAF was 0.0093. In terms of breast and ovarian cancer, odds ratios (ORs) were 0.41–1.53 (Table 4). Among studies investigating only ovarian cancer patients, the ORs were found to be similar, only Stafford et al. (2017) reported a significantly higher OR (OR: 4.95; *p* = 0.01; four of 48) [16]. In familial pancreatic cancer, lung cancer and upper aero-digestive tract (UADT) cancer, the carrier status meant a high odds ratio (4.24, 3 and 2.53, respectively) for developing cancer [8,10,17]. In the study of Akbari et al. (2008), c.9976A>T carrier status was associated with a high OR for developing esophageal squamous cell carcinoma (6.0; 95%CI: 1.3–28; *p* = 0.01) [27]. In other studies, *BRCA2* c.9976A>T carrier status was associated with a moderate risk for cancer (Table 4). 

Regarding the pathogenicity of the c.9976A>T variant, the effect of linkage with the *BRCA2* c.6275_6276delTT variant has been previously raised. Data of nine studies were available regarding the status of the *BRCA2* c.6275_6276delTT variant by analyzing 12,608 patients, including our results (Table 5). In one of the three cohorts reported by Higgs et al. (2015) and in the study by Meeks et al. (2016), increased carrier status of the deleterious variant (25/1576 and 233/306, respectively) besides c.9976A>T was described [13,15]. Excluding these two studies among the remaining 12,232 cancer patients, c.9976A>T and c.6275_6276delTT co-carrier status was reported only in 50 cases (0.4%).

## 3. Discussion

The clinical relevance of *BRCA2* C-terminal stop codon variants remains controversial. The *BRCA2* c.10095delinsGAATTATATCT variant located at the 3′ end of the gene is considered to be non-pathogenic based on the ENIGMA classification system. There are literature data regarding its allelic frequency and clinical relevance. Despite its low prevalence in control populations and its relatively higher frequency in breast/ovarian cancer patients, based on our and others’ findings (LOH, allele imbalance, segregation and linkage data), this variant can be considered as clinically non-significant.

*BRCA2* c.9976A>T, despite being a truncating variant, is usually classified as non-pathogenic based on case–control studies [11]. Indeed, in our study, the disease onset, tumor proliferation index or other pathological and clinical parameters did not differ in carriers compared to pathogenic *BRCA2* carriers or to *BRCA1/2* wild type patients. Additionally, we did not find an increased prevalence among carriers or in carrier families for HBOC. The MAF of c.9976A>T is around 1% among patients that also counts against its independent pathogenic role. The lack of genotype–phenotype segregation, lack of LOH and lack of allelic imbalance in patients are all in line with previous literature [11]. However, environmental factor-associated cancers (lung and skin carcinoma) were more frequent in families of the *BRCA2* c.9976A>T carrier probands.

In previously published data, breast cancer risk was mildly elevated in *BRCA2* c.9976A>T carriers when compared to control populations in three reports [9,14,15]. Studies investigating only ovarian cancer patients, except that of Stafford et al. (2017) [16], showed similar ORs. In the study of Stafford et al., in all cases, the germline c.9976A>T variant coexisted with other deleterious variants in other genes belonging to the *BRCA2* pathway. Among familial pancreatic, lung and upper aero-digestive tract (UADT) cancer patients, the c.9976A>T carrier status meant high odds (4.24, 3 and 2.53, respectively) for developing cancer [8,10,17]. In line with this, we observed an increased proportion of pancreatic cancer prevalence in families of double heterozygotes (c.9976A>T with pathogenic *BRCA1* variant), however, due to the limited number of cases, this observation should be validated in a larger sample cohort. Additionally, regarding pancreatic cancer, further analysis is subject to bias due to the secondary assessment of datasets. Others also suggested that the concomitant c.9976A>T variant should be considered during genetic counseling for a potentially earlier age of HBOC cancer onset [16,54,55]. In the study of Akbari et al. (2008), c.9976A>T carrier status was associated with a high OR of developing esophageal cancer (6.0; 95%CI: 1.3–28; *p* = 0.01) [27]. Higgs et al. (2015) also reported multiple co-occurrences of the *BRCA2* c.9976A>T variant with the pathogenic *BRCA2* c.6275_6276delTT (p.(Leu2092ProfsTer7)) frameshift variant in breast and ovarian cancer patients [13]. The authors concluded that associations of increased cancer risk due to *BRCA2* c.9976A>T represented a reporting bias and this was due to the variant being in linkage with *BRCA2* c.6275_6276delTT. However, in our patient cohort, neither investigated C-terminal stop codon variant was associated with any pathogenic *BRCA2* variant. Hence, we suggest that the linkage of the two *BRCA2* variants can be a founder phenomenon in the investigated cohort reported by Higgs et al. [13]. This is supported by other studies too [15], therefore, the reported variant associations may be a population specific-phenomenon representing a founder effect.

Although, based on our findings and previously published data, the *BRCA2* c.9976A>T variant alone probably cannot be considered as a risk factor for breast and ovarian cancer, it seems to be associated with other cancer types. Genetic epidemiological evidence suggested that the *BRCA2* c.9976A>T variant contributes to the risk of developing familial pancreatic cancer [8] and lung cancer [10,49,51]. Additionally, it was reported that the risk of developing lung cancer is approximately doubled for smokers compared to non-smokers when carrying the c.9976A>T variant [17,49]. Therefore, Wang et al. suggested that this finding may have implications for identifying high-risk ever-smoking subjects for lung cancer screening. Furthermore, it was reported [51] that the c.9976A>T variant was associated with cancers that have strong environmental genotoxic risk factors. Based on functional studies, the authors proposed that the variant protein could probably retain the DNA repair capabilities important to hormone-responsive tissues but it might be less efficient in counteracting genotoxic stress [51]. In line with this, based on associations between this *BRCA2* variant and upper aero-digestive tract and lung cancer risk, PARP1 inhibitors were suggested as potential treatment strategies [17,49]. These findings have not been confirmed by functional studies investigating the role of the c.9976A>T variant. Its damaging effects on the protein subcellular localization, cell viability, homology-directed repair (HDR) of double-strand breaks, centrosome amplification or sensitivity to DNA damaging agents [56,57] were not observed. Moreover, it has been suggested that the protein, translated from the variant-carrier transcript, is defective in maintaining fork stability while being still proficient in HDR. Therefore, c.9976A>T carriers may be insensitive to PARP inhibitors, which specifically exploits the defect of double-strand repair [7]. As a consequence, PARP-targeting therapy may not only be ineffective in these cases, but also induce further mutagenesis and genomic instability [7]. All these findings indicate that the clinical value of the use of PARP inhibitors in *BRCA2* c.9976A>T carriers should be further investigated.

In summary, the clinical phenotypes associated with C-terminal *BRCA2* variants are significantly different from those observed in families with highly penetrant *BRCA2* mutations [58,59]. For the expected pathogenic *BRCA2* mutation-associated cancer types (including breast, ovarian and prostate cancer), the C-terminal *BRCA2* variants have not been found as risk factors [49,59]. However, these variants may be involved in the pathogenesis of pancreatic and environmental factor-associated cancers.

## 4. Materials and Methods 

### 4.1. Cases: Patients and Relatives

We investigated 2491 independent patients (probands) with breast and/or ovarian cancer sent for germline *BRCA1/2* genetic analysis to the Department of Molecular Genetics at the National Institute of Oncology, Hungary between 2014–2019. Only one variant carrier per family, the proband, was included in our analysis. Among them, the *BRCA2* C-terminal stop codon variants (LRG_293t1:c.9976A>T and/or c.10095delinsGAATTATATCT) were identified in 49 cases (average age: 43.4±10.1 years; 47 females, 2 males). Estrogen, progesterone, HER2 receptor status, Ki67 proliferation indices and histology were assessed as part of the routine diagnostics. All data were collected from the institutional medical information system. Details (patient characteristics and histology findings) are summarized in Table 1. The study was approved by the Scientific and Research Committee of the Medical Research Council of the Ministry of Health, Hungary (ETT-TUKEB 53720-4/2019/EÜIG). Fisher’s exact test was used to examine the significance of the association (contingency) between phenotype and variant carrier status.

As a part of the Consortium of Investigators of Modifiers of *BRCA1/2* (CIMBA) and the Breast Cancer Association Consortium (BCAC), standard phenotypic and epidemiological data collection was applied from 1998 during the study (http://cimba.ccge.medschl.cam.ac.uk/ (accessed on 30 December 2020); http://bcac.ccge.medschl.cam.ac.uk/bcacdata/ (accessed on 30 December 2020)). Details of data collection protocols have been used and reported previously [60,61]. Accordingly, for the analysis of phenotypic and pedigree data, standard questionnaires (one is patient/disease-centered and one for pedigree data) were sent out to all patients in advance. Based on the standard data, acquisition pedigrees were generated. During genetic counseling, data reliability was confirmed by reviewing all medical reports available by practicing clinical geneticists. In the family history analysis, three-generation pedigrees were investigated where only the presence of tumor types were considered (not the number of cases in each family).

### 4.2. Nucleic Acid Extraction

Germline variants were analyzed using total DNA extracted from peripheral blood using a Gentra Puregene Blood Kit (Cat No.: 158389, Qiagen, Hilden, Germany) following the manufacturer’s instructions. 

A GeneRead DNA FFPE Kit (Cat No.: 180134, Qiagen, Hilden, Germany) was applied to isolate genomic DNA from formalin-fixed paraffin-embedded (FFPE) tissues in an automated way using the QIAcube Instrument (Qiagen, Hilden, Germany).

RNA extraction was performed from total blood taken into Tempus™ Blood RNA Tubes (Thermo Fisher Scientific, Waltham, MA, USA) by a Tempus™ Spin RNA Isolation Kit. RNA quality and quantity were determined by a NanoDrop^®^ 1000 Spectrophotometer (NanoDrop Technologies, Thermo Fisher Scientific, Waltham, MA, USA).

### 4.3. Genetic Analysis (Sequence and Copy Number Analysis by Next Generation Sequencing (NGS) and Multiplex Ligation-Dependent Probe Amplification)

Genetic analyses were done as we previously reported [62]. Germline *BRCA1/2* variant status was evaluated following library preparation using CE-IVD BRCA MASTR Plus Dx kit (Agilent, Santa Clara, CA, United States). Sequencing of the library was run on an Illumina MiSeq Instrument using MiSeq Reagent Kit v2 (500-cycles) (MS-102-2003, Illumina). Data analysis was done by MASTR Reporter software, a comprehensive CE-IVD marked (complies with the European In-Vitro Diagnostic Devices Directive) molecular solution for the identification of coding region variants in the *BRCA1* and *BRCA2* genes. Copy number analysis was performed by the Multiplex Ligation Dependent Probe Amplification (MLPA) method using P002 and P239 probe sets for *BRCA1*, and the P045 probe set for *BRCA2* (MRC-Holland, the Netherlands).

Clinical significance of variants was evaluated and interpreted following AMCG/AMG recommendations [1], ENIGMA classification [2] and literature data mining.

The following transcripts were used for variant annotation. *BRCA1*: LRG_292t1 (NM_007294.3) and *BRCA2*: LRG_293t1 (NM_000059.3).

### 4.4. Sanger Validation and LOH Analysis

All germline pathogenic, likely pathogenic and variants of unknown significance (VUSs) were validated by traditional bidirectional Sanger sequencing on an independent blood sample. For loss-of-heterogeneity (LOH) testing, DNA from tumor tissues was used for PCR amplification by a Qiagen Multiplex PCR Kit (Qiagen). PCR product was purified by ExoSAP-IT™ reagents (Thermo Fisher Scientific, Waltham, MA, United States), then purified amplicons were sequenced bidirectionally on an ABI3130 Genetic Analyzer (Applied Biosystems, Thermo Fisher Scientific, Waltham, MA, USA) using a BigDye™ Terminator v.1.1 kit (Thermo Fisher Scientific, Waltham, MA, USA).

### 4.5. Transcript Allelic Imbalance

Relative expression of the variant carrier and the normal allele was tested with Sanger sequencing. The ratio of electropherograms of the variant position on the cDNA template relative to the gDNA template was calculated. Briefly, cDNA was generated from 500 ng RNA using SuperScript™ IV Reverse Transcriptase (Thermo Fisher Scientific). cDNA primers were designed using Primer3Plus software (https//:primer3plus.com): B2-C-e24_For—GATCCAGACTTTCAGCCATCTT and Rd_B2_Ex27.01_Rev—CGTCGTTTCAGTCTGAGATAATCT. Following PCR amplification and Sanger sequencing, data were visualized in Sequence Scanner software (Applied Biosystems, Thermo Fisher Scientific), and the peak ratio of the heterozygote position was given and compared to the peak ratio of the gDNA sequence of the same position for the same sample. The relative ratio was calculated and allelic imbalance was declared if the difference was >50%.

### 4.6. Statistical Analysis

For both proband characterization and family description, proportions and 95% confidence intervals by a modified Wald method were calculated using GraphPad QuickCalcs (https://www.graphpad.com/quickcalcs/confInterval1/ (accessed on 30 December 2020)). For statistical analysis, 2 × 2 contingency tables were applied and *p* values were calculated by Fisher’s exact test. *p* values were considered statistically significant at <0.05. Statistical power was calculated using the ClinCalc online algorithm (https://clincalc.com/stats/samplesize.aspx (accessed on 30 December 2020)).

## 5. Conclusions

As a conclusion, our results suggest that among *BRCA2* C-terminal stop codon variants, c.10095delinsGAATTATATCT is clinically non-significant. However, the c.9976A>T variant may have different clinical significance compared to the *BRCA2* truncating variant before amino acid 3326. It may be considered as a genetic modifying factor in pancreas cancer when it co-occurs with pathogenic *BRCA1* variants, although this observation should be validated in a larger sample cohort of double heterozygotes. Additionally, it seems to have an impact on the development of tumor types where environmental factors are significant as a genotoxic stress factor. Therefore, it is suggested to be a non-negligible variant, especially in the risk assessment of environmental cancers. The ACMG “pathogenic” classification is disease-specific. That is, a variant classified as (likely) benign with respect to HBOC still cannot be disregarded in conjunction with other, only loosely associated, diseases or with possible treatment options.

Additionally, our data, in line with a very recent review [63], suggest that collecting disease-specific clinical data regarding C-terminal *BRCA2* variants can assist in reducing the number of VUSs, which in turn may help in more precise treatment planning.

## Figures and Tables

**Table 1:** Patient characteristics harboring *BRCA2* C-terminal variants (LRG_292t1:c.9976A>T, p.(Lys3326Ter) and LRG_292t1:c.10095delinsGAATTATATCT, p.(Ser3366AsnfsTer4)).

					Proband								
#	Gender	*BRCA2* C-Terminal Variant	Pathogenic *BRCA1/BRCA2* Variant	Disease:	1st Breast Cancer						2nd Breast Cancer	Ovarian Cancer	
					Age of onset	ER	PR	HER2	Ki67 (%)	Hist	Age	Age	Hist
1	F	c.9976A>T	—	sol	43	pos	pos	neg	25	DUC	-	-	-
2	F	c.9976A>T	—	sol	55	pos	neg	neg	n.a.	DUC	-	-	-
3	F	c.9976A>T	—	sol	48	pos	pos	neg	n.a.	DUC	-	-	-
4	F	c.9976A>T	—	sol	47	pos	pos	neg	5	DUC	-	-	-
5	F	c.9976A>T	—	sol	31	pos	pos	neg	40	DUC	-	-	-
6	F	c.9976A>T	—	sol	68	pos	pos	neg	1	DUC	-	-	-
7	F	c.9976A>T	—	sol	57	pos	pos	neg	10	DUC	-	-	-
8	F	c.9976A>T	—	sol	36	neg	neg	neg	40	DUC	-	-	-
9	F	c.9976A>T	—	sol	42	pos	pos	neg	70	LOB	-	-	-
10	F	c.9976A>T	—	sol	46	pos	pos	neg	n.a.	DUC	-	-	-
11	F	c.9976A>T	—	sol	41	pos	pos	pos	10	DUC	-	-	-
12	F	c.9976A>T	—	sol	34	pos	pos	pos	67	DUC	-	-	-
13	F	c.9976A>T	—	sol	39	neg	neg	neg	20	DUC	-	-	-
14	F	c.9976A>T	—	sol	46	pos	pos	neg	5	DUC	-	-	-
15	F	c.9976A>T	—	sol	41	pos	neg	neg	25	DUC	-	-	-
16	F	c.9976A>T	—	sol	46	pos	pos	neg	n.a.	DUC	-	-	-
17	F	c.9976A>T	—	sol	44	pos	pos	neg	n.a.	DUC	-	-	-
18	F	c.9976A>T	—	sol	38	pos	pos	neg	25	DUC	-	-	-
19	F	c.9976A>T	—	multi	33	pos	pos	neg	50	DUC	-	33	adenocar-cinoma
20	F	c.9976A>T	—	sol	33	pos	pos	neg	25	DUC	-	-	-
21	F	c.9976A>T	—	sol	61	neg	neg	neg	25	DUC	-	-	-
22	F	c.9976A>T	—	sol	46	neg	neg	poz	10	DUC	-	-	-
23	F	c.9976A>T	—	sol	40	pos	neg	neg	70	DUC	-	-	-
24	F	c.9976A>T	—	multi	48	pos	n.a.	neg	n.a.	DUC	48	-	-
25	F	c.9976A>T	—	sol	26	pos	pos	pos	35	DUC	-	-	-
26	F	c.9976A>T	—	sol	41	pos	pos	neg	n.a.	DUC	-	-	-
27	F	c.9976A>T	—	sol	44	pos	pos	neg	25	DUC	-	-	-
28	F	c.9976A>T	—	sol	33	pos	pos	neg	35	DUC	-	-	-
29	F	c.9976A>T	—	sol	43	pos	pos	neg	7	DUC	-	-	-
30	M	c.9976A>T	—	sol	27	n.a.	n.a.	n.a.	n.a.	DUC	-	-	-
31	F	c.9976A>T	—	sol	n.a.	n.a.	n.a.	n.a.	n.a.	n.a.	-	45	cystadenoc arcinoma mucinosum
32	F	c.10095delinsGAATTATATCT	—	sol	44	pos	pos	neg	10	DUC	-	-	-
33	F	c.10095delinsGAATTATATCT	—	sol	36	neg	neg	neg	30	DUC	-	-	-
34	F	c.10095delinsGAATTATATCT	—	sol	55	neg	neg	neg	50	DUC	-	-	-
35	F	c.10095delinsGAATTATATCT	—	sol	55	n.a.	n.a.	n.a.	n.a.	n.a.	-	-	-
36	F	c.10095delinsGAATTATATCT	—	sol	40	pos	pos	neg	15	DUC	-	-	-
37	F	c.10095delinsGAATTATATCT	—	sol	27	pos	pos	neg	n.a.	LOB	-	-	-
38	F	c.10095delinsGAATTATATCT	—	sol	39	pos	pos	neg	10	LOB	-	-	-
39	F	c.10095delinsGAATTATATCT	—	sol	45	pos	pos	neg	1	DUC	-	-	-
40	F	c.10095delinsGAATTATATCT	—	sol	38	pos	pos	neg	2	DUC	-	-	-
41	F	c.10095delinsGAATTATATCT	—	multi	55	pos	pos	neg	20	LOB	58	-	-
42	F	c.9976A>T & c.10095delinsGAATTATATCT	—	sol	41	pos	pos	neg	n.a.	DUC	-	-	-
43	F	c.10095delinsGAATTATATCT	*BRCA1* c.5251C>T (p.Arg1751*)	sol	n.a.	n.a.	n.a.	n.a.	n.a.	n.a.	-	36	high grade serosus carcinoma
44	F	c.9976A>T	*BRCA1* c.1687C>T (p.Gln563*)	sol	40	pos	pos	pos	50	DUC	-	-	-
45	F	c.9976A>T	*BRCA1* c.68_69delAG (p.Glu23Valfs*)	sol	54	neg	neg	neg	85	DUC	-	-	-
46	F	c.9976A>T	*BRCA1* c.3018_3021del4 (p.His1006Glnfs*17)	sol	38	n.a.	n.a.	n.a.	n.a.	n.a.	-	-	-
47	F	c.9976A>T	*BRCA1* c.181T>G (p.Cys61Gly)	sol	49	neg	neg	neg	90	DUC	-	-	-
48	M	c.9976A>T	*BRCA2* c.8378G>A (p.Gly2793Glu)	sol	79	pos	pos	neg	25	DUC	-	-	-
49	F	c.10095delinsGAATTATATCT	*BRCA2* c.7595_7596insTT (p.Ala2534Leufs*18)	sol	38	poz	poz	n.a.	5	DUC	-	-	-

Sol: solitaire (breast or ovarian cancer only); multi: multiple (two breasts or breast and ovarian cancer); ER: estrogen receptor; PR: progesterone receptor; HER2: human epidermal growth factor receptor 2; Ki67: proliferation index; pos: positive immunohistochemistry; neg: negative immunohistochemistry; DUC: invasive ductal carcinoma or ductal carcinoma in situ, LOB: invasive lobular carcinoma; n.a.: not available, Hist: histology.

**Table 2:** Cancer prevalence and tumor characteristics in hereditary breast and ovarian cancer (HBOC) probands and their families (proportions with ± 95%CI).

Clinicopathological Parameter	*BRCA1/2* Wild Type	Pathogenic *BRCA1* Variant	Pathogenic *BRCA2* Variant	*BRCA2* c.9976A>T	*BRCA2* c.10095delins GAATTATATCT	Pathogenic *BRCA1* + *BRCA2* c.9976A>T
Number of probands (*n*)	1947	307	188	31	10	4
Age at disease onset (years):						
Breast cancer (mean ± SD)	43.38 ± 9.33	39.48 ± 8.93 **a**	41.93 ± 8.63 **b**	43.1 ± 9.10 **b**	43.4 ± 9.37	42.25 ± 7.54
Ovarian cancer (mean ± SD)	49.07 ± 13.85	48.41 ± 7.99	55.3 ± 10.03	45.00	—	—
Male breast cancer (mean ± SD)	59.74 ± 11.08	47.00	62.87 ± 8.82	27.00	—	—
Multiplex tumors from all patients (proportion (mpx cases/all))	0.08 (156/1947)	0.2 (60/307) **a**	0.18 (34/188) **a**	0.06 (2/31)	0.10 (1/10)	0.00 (0/4)
Tumor characteristics:						
Ki67 of breast cancer (mean ± SD)	30 ± 25	58 ± 24 **a**	30 ± 24	28 ± 21	17 ± 16	75 ± 22
ER pos proportion (95%CI)	0.70 (0.68–0.72)	0.19 (0.15–0.24) **a**	0.78 (0.72–0.84) **b**	0.86 (0.69–0.95) **b**	0.77 (0.44–0.95) **b**	0.33 (0.05–0.79)
PR pos proportion (95%CI)	0.64 (0.62–0.66)	0.15 (0.11–0.20) **a**	0.67 (0.60–0.74) **b**	0.75 (0.56–0.87)	0.77 (0.44–0.94)	0.33 (0.05–0.79)
HER2 pos proportion (95%CI)	0.24 (0.22–0.26)	0.06 (0.04–0.11) **a**	0.10 (0.06–0.16) **a**	0.13 (0.05–0.31)	0 (0.00–0.34)	0.33 (0.05–0.79)
TNBC proportion (95%CI)	0.20 (0.19–0.23)	0.75 (0.70–0.80) **a**	0.19 (0.14–0.26) **b**	0.10 (0.03–0.27) **b**	0.22 (0.05–0.55)	0.66 (0.20–0.94)
Tumor prevalence in families (proportion (95%CI))						
Breast cancer <50 years of age in the family	0.10 (0.09–0.11)	0.21 (0.16–0.25) **a**	0.24 (0.18–0.30) **a**	0.16 (0.06–0.33)	0.10 (0.00–0.42)	0 (0.00–0.54)
Breast cancer at any age in the family	0.42 (0.40–0.44)	0.61 (0.56–0.66) **a**	0.60 (0.53–0.67) **a**	0.38 (0.24–0.56) **b,c**	0.5 (0.24–0.76)	0.25 (0.03–0.71)
Ovarian cancer at any age in the family	0.06 (0.05–0.08)	0.21 (0.17–0.26) **a**	0.08 (0.05–0.13) **b**	0.06 (0.01–0.21)	0 (0.00–0.32)	0.25 (0.03–0.71)
Breast and/or ovarian cancer at any age in the family	0.47 (0.44–0.49)	0.69 (0.63–0.74) **a**	0.65 (0.58–0.72) **a**	0.45 (0.29–0.62) **b,c**	0.5 (0.23–0.76)	0.5 (0.15–0.85)
Prostate cancer in the family	0.08 (0.07–0.09)	0.04 (0.03–0.07) **a**	0.11 (0.07–0.17) **b**	0.06 (0.01–0.21)	0.20 (0.05–0.52)	0.25 (0.03–0.71)
Pancreatic cancer in the family	0.04 (0.03–0.05)	0.06 (0.04–0.09)	0.10 (0.06–0.15) **a**	0.09 (0.03–0.25)	0 (0.00–0.32)	0.5 (0.15–0.85) **a,b**
Lung cancer in the family	0.13 (0.12–0.15)	0.09 (0.06–0.13)	0.09 (0.05–0.14)	0.22 (0.11–0.40) **b,c**	0 (0.00–0.32)	0 (0.00–0.54)
Skin cancer in the family	0.04 (0.03–0.05)	0.03 (0.02–0.06)	0.03 (0.01–0.07)	0.09 (0.02–0.25)	0.10 (0.00–0.42)	0 (0.00–0.54)
Head and neck cancer in the family	0.05 (0.04–0.06)	0.05 (0.03–0.08)	0.08 (0.05–0.13)	0 (0.00–0.13)	0 (0.00–0.32)	0 (0.00–0.54)
Hepatobiliary cancer in the family	0.03 (0.02–0.04)	0.03 (0.01–0.05)	0.04 (0.02–0.08)	0 (0.00–0.13)	0 (0.00–0.32)	0.25 (0.03–0.71)
Gastric cancer in the family	0.08 (0.07–0.09)	0.08 (0.06–0.12)	0.03 (0.01–0.06)	0.09 (0.02–0.25)	0.10 (0.00–0.42)	0 (0.00–0.54)

TNBC: triple-negative breast cancer. Significance (*p* < 0.05 based on Fisher’s exact *t*-test) is indicated by letters, where “a”: compared to wild type; “b”: compared to *BRCA1*; “c”: compared to *BRCA2*. Relevant associations with *BRCA2* c.9976A>T are highlighted with bold letters.

**Table 3:** Prevalence of *BRCA2* c.10095delinsGAATTATATCT based on literature data.

Cancer Type	Reference	Number of Probands Screened (Germline)	Number of Patients Carrying *BRCA2* c.10095delinsGAATTATATCT Variant	Allelic Frequency	Clinical Interpretation
breast, ovarian cancer	Meindl et al. 2002 [20]	989	3	0.00303	VUS
breast, ovarian cancer	Ratajska et al. 2008 [18]	64	2	0.03125	VUS
breast, ovarian cancer	Machackova et al. 2008 [19]	1010	1	0.00099	VUS
breast, ovarian cancer	Cvok et al. 2008 [21]	115	1	0.00869	clinically not important
breast, ovarian cancer	Thomassen et al. 2008 ** [25]	na	1	na	not interpreted
breast, ovarian cancer	Meisel et al. 2017 **^,†^ [26]	523	3	0.00573	VUS
ovarian cancer	Koczkowska et al. 2016 [23]	22*	1	na *	benign
pancreatic cancer	Hahn et al. 2013 [22]	26	1	0.03846	VUS

*: Of all samples harboring pathogenic somatic *BRCA1/2* variants, 22 were selected for further germline testing, therefore allelic frequency in the investigated cohort cannot be estimated; **: the whole genotype of each sample is not known due to the application of a denaturing high-performance liquid chromatography (DHPLC) screening method before sequencing; ^†^: it is not clarified if c.10095delinsGAATTATATCT was detected individually or associated with other pathogenic *BRCA1/2* variants. VUS: variant of unknown significance; na: not available.

**Table 4:** Prevalence and association of *BRCA2* c.9976A>T variant with various cancers in 38 studies.

Cancer Type	Reference	Number of Probands Screened	Number of Patients Carrying *BRCA2* c.9976A>T Variant	Allelic Frequency	Odds Ratio (OR) (Patients vs. Controls) (Confidence Intervals)
breast, ovarian cancer	current study	2138	46	0.01485	na
breast cancer	Mazoyer et al. 1996 [11]	513	11	0.01267	OR: 1.01 (0.41–2.48)
breast cancer	Johnson et al. 2007 [12]	473	11	0.011628	OR: 1.16 (0.79–1.63)
breast cancer	Borg et al. 2010 [28]	2103	40	0.00951	na
breast cancer	Michailidou et al. 2013 [14]	10052	80	0.008	RR: 1.39 1.39 (1.13–1.71)
breast cancer	Thompson et al. 2015 [9]	2634	66	0.01252	OR: 1.53 (1.00–2.34); (*p* = 0.047)
breast cancer	Meeks et al. 2016 [15]	41081	852	0.01036	OR: 1.28 (1.17–1.40); (*p* = 5.86 × 10^−6^)
breast cancer, early onset	Krainer et al.l. 1997 [29]	73	1	0.00684	na
breast cancer, early onset	Malone et al. 2000 [30]	386	2	0.00259	na
breast cancer, early onset	Bergthorsson et al. 2001 [31]	119	1	0.00420	na
breast cancer, early onset	Hamann et al. 2003 [32]	91	1	0.00549	na
breast cancer, early onset	Musolino et al. 2007 [33]	66	3	0.02272	na
breast cancer, early onset	Juwle et al. 2012 [34]	50	2	0.01	na
breast cancer, early onset	Juwle et al. 2012 [34]	50	2	0.02	na
breast, ovarian cancer	Claes et al. 2003 [35]	249	8	0.01606	na
breast, ovarian cancer	Hadjisavvas et al. 2003 [36]	26	1	0.01923	na
breast, ovarian cancer	Giannini et al. 2006 [37]	73	1	0.00684	na
breast, ovarian cancer	Simard et al. 2007 [38]	143	2	0.00699	na
breast, ovarian cancer	Beristain et al. 2007 [39]	236	1	0.00211	na
breast, ovarian cancer	Ratajska et al. 2008 [18]	64	0	0	na
breast, ovarian cancer	Kuusisto et al. 2011 [40]	82	1	0.012	OR: 0.41 (0.05–3.24); (*p* = 0.702)
breast, ovarian cancer	Cherbal et al. 2012 [41]	79	1	0.00632	na
breast, ovarian cancer	Jalkh et al. 2012 [42]	72	1	0.00694	na
breast, ovarian cancer	Dobričić et al. 2013 [43]	71	1	0.00704	na
breast, ovarian cancer	Higgs et al. 2015-cohort 1 [13]	1850	23	0.00621	
breast, ovarian cancer	Higgs et al. 2015-cohort 2 [13]	1576	not reported	na	na
breast, ovarian cancer	Higgs et al. 2015-cohort 3 [13]	1395	43	0.01541	
ovarian cancer	Mazoyer et al. 1996 [11]	361	7	0.00969	na
ovarian cancer	Hilton et al. 2002 [44]	92	1	0.00543	na
ovarian cancer	Meeks et al. 2016 [15]	14514	311	0.01071	OR: 1.26 (1.10–1.43); (*p* = 3.84 ×10^−3^)
ovarian cancer	Stafford et al. 2017 [16]	48	4*	0.00416	OR: 4.95 (*p* = 0.01)
male breast	Haraldsson et al. 1998 [45]	34	1	0.01470	na
male breast	Ding et al. 2011 [46]	115	2	0.00869	na
male breast	Evans et al. 2008 [47]	64	1	0.00781	na
familial pancreatic cancer	Martin et al. 2005 [8]	144	8	0.02777	OR: 4.24 (*p* < 0.05)
sporadic pancreatic cancer	Obazee et al. 2019 [48]	2835	69	0.0123	OR: 1.78 (1.26–2.52); (*p* = 0.00119)
lung cancer	Wang et al. 2014 [49]	21435	298	0.01434	OR: 1.83 (OR) = 2.47; (*p* = 4.74 × 10^−20^)
lung cancer	Rudd et al. 2006 [50]	1526	14	0.009	OR: 1.72 (0.15–2.57); (*p* = 0.0075)
lung cancer	Rafnar et al. 2018 [51]	4 461	na	na	OR: 1.54 (1.23–1.91); (*p* = 0.00012)
*incl:* small cell lung cancer		800	na	na	OR: 2.06 (1.35–3.16)
*incl:* squamous cell lung carcinoma (SQLC)		901	na	na	OR: 1.71 (1.10–2.67); (*p* = 0.02)
lung squamous cell carcinoma	Esai Selvan et al. 2019 [10]	318	na	na	OR: 3.0 (1.4–6.4); (*p* = 0.0053)
esophageal squamous cell carcinoma	Akbari et al. 2008 [27]	197	9	0.02284	OR: 6.0 (1.3–28);(*p* = 0.01)
UADT squamous cell carcinoma	Delahaye-Sourdeix et al. 2015 [17]	5942	149	0.01253	OR: 2.53 (1.89–3.38); (*p* = 3 × 10^−10^)
bladder cancer	Ge et al. 2016 [52]	3591	41	0.0096	OR: 1.70 (1.19–2.42); (*p* = 0.0036)
renal cell carcinoma	Ge et al. 2016 [52]	1322	13	0.0125	OR: 1.60 (0.91–2.82); (*p* = 0.103)
prostate cancer	Ge et al. 2016 [52]	1151	8	0.0076	OR: 0.85 (0.41–1.74); (*p* = 0.647)
squamous cell carcinoma of the skin	Rafnar et al. 2018 [51]				OR: 1.69 (1.26–2.26)
melanoma	Tuominen et al. 2016 [53]	452	12	0.01304	OR: 2.80 (1.04–7.58), (*p* = 0.035)

SQLC: squamous cell lung carcinoma; UADT: upper aero-digestive tract.

**Table 5:** Studies analyzing linkage of c.9976A>T and c.6275_6276delTT.

Study	Note		Number of Cases Screened	Number of Cases with *BRCA2* c.9976A>T Variant Alone	Number of Cases Carrying *BRCA2* c.9976A>T Variant WITH *BRCA2* c.6275_6276delTT Variant	
					(#)	(%)
Current study		breast, ovarian cancer	2138	46	0	0
Higgs et al. 2015 [13]	High-risk breast/ovarian cancer families, Manchester region of North West England	breast, ovarian cancer	1850	23	18	0.0097
	Research study: familial breast/ovarian cancer cases, North West	breast, ovarian cancer	1576	not reported	25	0.0159
	Samples from Liverpool (UK), Irish Republic, Finland and Germany	breast, ovarian cancer	1395	43	4	0.0029
Mazoyer et al. 1996 [11]		breast cancer	513	11	2	0.0039
Mazoyer et al. 1996 [11]		ovarian cancer	361	7	0	0
Martin et al. 2005 [8]		familial pancreatic cancer	144	8	0	0
Akbari et al. 2008 [27]		esophageal squamous cell carcinoma	197	9	0	0
Wang et al. 2014 [49]	Meta-analysis of 4 lung cancer GWAS studies	lung cancer	21,435	298	0/70	0
Rafnar et al. 2018 [51]	Analysis of 3 studies	lung cancer	4461	na	0	0
		*incl:* small cell lung cancer	800	na	0	0
		*incl:* squamous cell lung carcinoma (SQLC)	901	na	0	0
Meeks et al. 2016 [15]		breast cancer	41,081	852	233/306	0.7614
Haraldsson et al. 1998 [45]		male breast cancer	34	0	1	0.0294

GWAS: genome wide association study.

## Data Availability

All relevant data are included in the manuscript.

## Supplementary Materials

The following are available online at https://www.mdpi.com/2072-6694/13/4/881/s1, Table S1: Family history of C-terminal stop codon carrier probands collected from three-generation pedigrees; Table S2: Supplementary Table 2. Fisher’s exact *p* values of comparison of tumor prevalence in families. Figure S1. Annotation of NM_000059.3(*BRCA2*):c.10095delinsGAATTATATCT (p.Ser3366Asnfs*4) variant. A: c.10095delinsGAATTATATCT is annotated as two different variants (insertions) in gnomAD: NM_000059.4(*BRCA2*):c.10095_10096insT (p.Ser3366Ter) and NM_000059.3(*BRCA2*):c.10094_10095insGAATTATAT (p.Ser3366_Glu3367insAsnTyrIle). B: Integrative Genomics Viewer (IGV) image of the “two misannotated variants”. The *cis* allelic position of the c.10094_10095insGAATTATAT and c.10095_10096insT variants are clearly visible on the IGV images of next generation sequencing data from gnomAD pages of both entries; they are always on the same reads of the pile-up IGV track and they were separated into their allelic primitives during variant calling.
