# Supplementary material for: Application of Multilayer Evidence for Annotation of C-Terminal BRCA2 Variants
Source: Cancers (Basel). 2021 Feb 20;13(4):881. doi: 10.3390/cancers13040881 (PMC7923782; doi:10.3390/cancers13040881)
Supplement: Supplementary file 1 [file cancers-13-00881-s001.pdf]

Application of Multilayer Evidence for Annotation of C-terminal BRCA2 Variants

Henriett Butz, János Papp, Anikó Bozsik, Lilla Krokker, Tímea Pócza, Edit Oláh and Attila Patócs

Table S1. Family history of C-terminal stop codon carrier probands collected from three generation pedigrees.

Patient #	Breast cancer <50 years of age in the family	Breast cancer at any age in the family	Ovarian cancer at any age in the family	Breast and/or ovarian cancer at any age in the family	Prostate cancer in the family	Pancreatic cancer in the family	Other cancer types in the family
1	no	yes	no	yes	no	no	colorectal
2	no	no	yes	yes	no	no	gastric, colorectal
3	no	yes	no	yes	no	no	lung
4	no	no	no	no	no	no	
5	yes	yes	no	yes	no	no	
6	no	yes	no	yes	no	no	
7	no	no	no	no	yes	no	
8	no	no	no	no	no	no	
9	no	no	no	no	yes	yes	
10	no	no	no	no	no	no	skin
11	yes	yes	no	yes	no	no	skin
12	no	no	no	no	no	no	gastric
13	no	no	no	no	no	no	lung
14	yes	yes	no	yes	no	no	lung
15	no	no	no	no	no	no	
16	no	no	yes	yes	no	no	uterus
17	no	no	no	no	no	yes	cervix
18	no	yes	no	yes	no	no	
19	no	no	no	no	no	no	
20	no	no	no	no	no	no	
21	yes	yes	no	yes	no	no	esophageal, lung
22	yes	yes	no	yes	no	yes	lung, skin
23	no	no	no	no	no	no	brain, lung
24	yes	yes	no	yes	no	no	
25	no	no	no	no	no	no	
26	no	yes	no	yes	no	no	
27	no	no	no	no	no	no	
28	no	yes	no	yes	no	no	
29	no	no	no	no	no	no	
30	no	no	no	no	no	no	lung
31	no	no	no	no	no	no	colorectal, gastric
32	no	yes	no	yes	no	no	
33	no	no	no	no	no	no	gastric
34	no	no	no	no	yes	no	skin, cervix
35	no	no	no	no	no	no	
36	no	no	no	no	no	no	
37	no	no	no	no	no	no	
38	no	yes	no	yes	no	no	

39	yes	yes	no	yes	no	no	
40	no	no	no	no	yes	no	
41	no	yes	no	yes	no	no	renal
42	no	no	no	no	no	no	
43	no	yes	no	yes	no	no	
44	no	no	yes	yes	no	no	
45	no	yes	no	yes	yes	no	hepatobiliary cancer
46	no	no	no	no	no	yes	brain
47	no	no	no	no	no	yes	
48	no	yes	no	yes	no	no	
49	yes	yes	no	yes	no	no	renal, colorectal, cervix

Table S2. p values of comparison of tumor prevalence in families using Fisher exact test. Wt: wild type *BRCA1* and *BRCA2*; Br: breast cancer; Ov: ovarian cancer; cc: cancer.

Groups:	Wt_vs_BRCA1	Wt_vs_BRCA2	Wt_vs_c.9976A>T	Wt_vs_c.10095delins11	Wt_vs_BRCA1+ c.9976A>T	BRCA1_vs_BRCA2	BRCA1_vs_c.9976A>T	BRCA1_vs_c.10095delins11	BRCA1_vs_BRCA1+ c.9976A>T	BRCA2_vs_c.9976A>T	BRCA2_vs_c.10095delins11	BRCA2_vs_BRCA1+ c.9976A>T	Lyster_vs_c.10095delins11	c.9976A>T_vs_BRCA1+ c.9976A>T	c.10095delins11_vs_BRCA1+ c.9976A>T
Br<50_yrs_of_age_yes	1.3675E-06	4.6994E-07	3.6525E-01	1.0000E+00	1.0000E+00	3.7241E-01	6.4639E-01	6.9321E-01	5.8614E-01	4.8829E-01	4.5833E-01	5.7464E-01	1.0000E+00	1.0000E+00	1.0000E+00
Br_any_age_yes	7.4918E-10	2.3345E-06	7.1753E-01	7.5208E-01	6.4115E-01	8.4971E-01	2.0060E-02	5.1838E-01	3.0293E-01	3.0191E-02	5.2393E-01	3.0397E-01	7.1426E-01	1.0000E+00	5.8042E-01
Ov_any_age_yes	2.6061E-13	3.6932E-01	1.0000E+00	1.0000E+00	2.4787E-01	1.6005E-04	5.7570E-02	2.2362E-01	1.0000E+00	1.0000E+00	1.0000E+00	3.1196E-01	1.0000E+00	3.1322E-01	2.8571E-01
Br/Ov_any_age_yes	4.0140E-13	1.2639E-06	8.5859E-01	1.0000E+00	1.0000E+00	4.2889E-01	9.2837E-03	2.9724E-01	5.9137E-01	4.4003E-02	3.2862E-01	6.1201E-01	1.0000E+00	1.0000E+00	1.0000E+00
Prostate cc_yes	3.5942E-02	9.7027E-02	1.0000E+00	1.9224E-01	2.8686E-01	4.0287E-03	6.4880E-01	8.4872E-02	1.8022E-01	5.4186E-01	3.4730E-01	4.0232E-01	2.4543E-01	3.1322E-01	1.0000E+00
Pancreatic cc_yes	3.0920E-01	2.3459E-03	1.6825E-01	1.0000E+00	1.1885E-02	1.1177E-01	4.2472E-01	1.0000E+00	2.1850E-02	1.0000E+00	6.0250E-01	5.9882E-02	5.6379E-01	8.8904E-02	6.5934E-02
Lung cc_yes	4.5000E-02	7.2556E-02	1.8362E-01	3.7521E-01	1.0000E+00	1.0000E+00	3.3581E-02	6.0755E-01	1.0000E+00	5.4859E-02	1.0000E+00	1.0000E+00	1.6446E-01	5.6203E-01	1.0000E+00
Skin cc_yes	6.3517E-01	8.4250E-01	1.2745E-01	3.3481E-01	1.0000E+00	1.0000E+00	1.0626E-01	3.0119E-01	1.0000E+00	1.1914E-01	3.0826E-01	1.0000E+00	1.0000E+00	1.0000E+00	1.0000E+00
Head and neck cc_yes	1.0000E+00	1.4173E-01	4.1353E-01	1.0000E+00	1.0000E+00	2.0011E-01	3.8349E-01	1.0000E+00	1.0000E+00	1.3617E-01	1.0000E+00	1.0000E+00	1.0000E+00	1.0000E+00	1.0000E+00
Hepatobiliary cc_yes	7.3770E-01	6.8183E-01	6.2512E-01	1.0000E+00	1.3791E-01	4.5364E-01	1.0000E+00	1.0000E+00	1.2311E-01	6.0455E-01	1.0000E+00	1.7601E-01	1.0000E+00	1.1429E-01	2.8571E-01
Gastric cc_yes	8.2623E-01	2.7644E-03	7.4306E-01	5.8874E-01	1.0000E+00	7.5629E-03	7.4659E-01	1.0000E+00	1.0000E+00	8.7785E-02	2.7025E-01	1.0000E+00	1.0000E+00	1.0000E+00	1.0000E+00

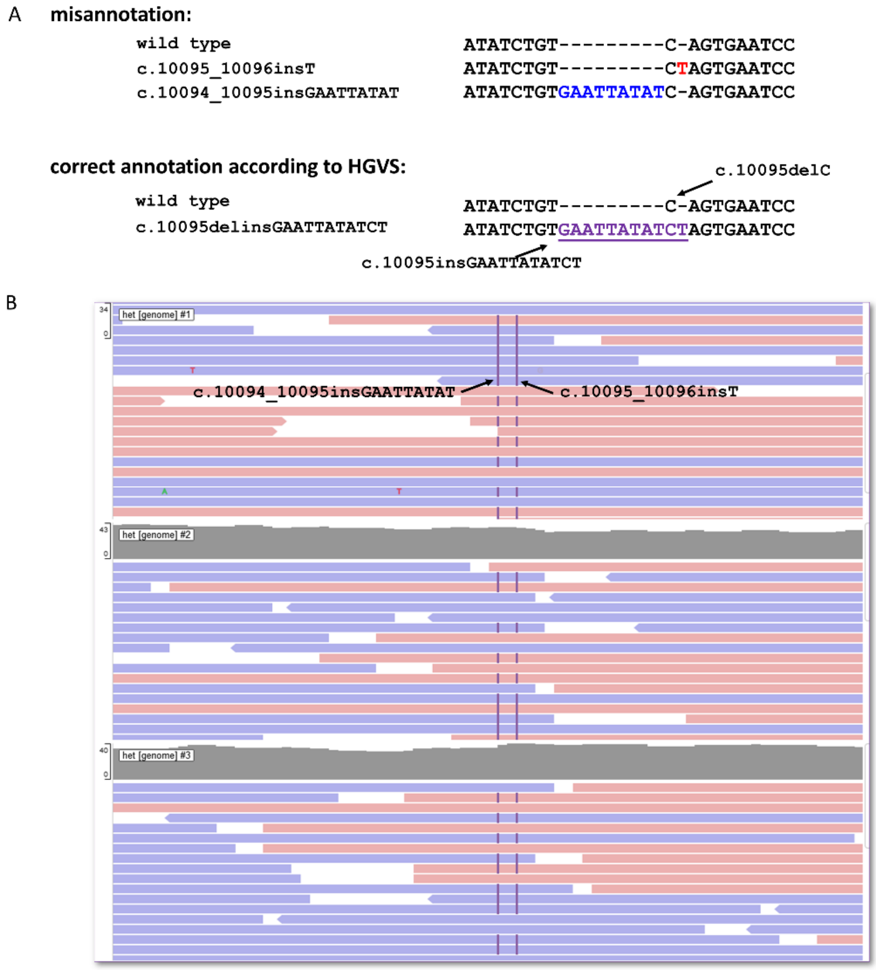

Figure S1. Annotation of the BRCA2 c.10095delinsGAATTATATCT.
